# DNAJB3 attenuates ER stress through direct interaction with AKT

**DOI:** 10.1371/journal.pone.0290340

**Published:** 2023-08-18

**Authors:** Zeyaul Islam, Abdoulaye Diane, Namat Khattab, Mohammed Dehbi, Paul Thornalley, Prasanna R. Kolatkar

**Affiliations:** Qatar Biomedical Research Institute (QBRI), Hamad Bin Khalifa University (HBKU), Qatar Foundation, Doha, Qatar; Northwest University, UNITED STATES

## Abstract

Metabolic stress involved in several dysregulation disorders such as type 2 diabetes mellitus (T2DM) results in down regulation of several heat shock proteins (HSPs) including DNAJB3. This down regulation of HSPs is associated with insulin resistance (IR) and interventions which induce the heat shock response (HSR) help to increase the insulin sensitivity. Metabolic stress leads to changes in signaling pathways through increased activation of both c-jun N-terminal kinase-1 (JNK1) and the inhibitor of κB inflammatory kinase (IKKβ) which in turn leads to inactivation of insulin receptor substrates 1 and 2 (IRS-1 and IRS-2). DNAJB3 interacts with both JNK1 and IKKβ kinases to mitigate metabolic stress. In addition DNAJB3 also activates the PI3K-PKB/AKT pathway through increased phosphorylation of AKT1 and its substrate AS160, a Rab GTPase-activating protein, which results in mobilization of GLUT4 transporter protein and improved glucose uptake. We show through pull down that AK T1 is an interacting partner of DNAJB3, further confirmed by isothermal titration calorimetry (ITC) which quantified the avidity of AKT1 for DNAJB3. The binding interface was identified by combining protein modelling with docking of the AKT1-DNAJB3 complex. DNAJB3 is localized in the cytoplasm and ER, where it interacts directly with AKT1 and mobilizes AS160 for glucose transport. Inhibition of AKT1 resulted in loss of GLUT4 translocation activity mediated by DNAJB3 and also abolished the protective effect of DNAJB3 on tunicamycin-induced ER stress. Taken together, our findings provide evidence for a direct protein-protein interaction between DNAJB3 and AKT1 upon which DNAJB3 alleviates ER stress and promotes GLUT4 translocation.

## 1. Introduction

Molecular chaperones play an important role in the maintenance of cellular proteostasis. These macromolecules include heat-shock proteins (HSPs) that are ubiquitous and assist in the recovery from stress either by repairing misfolded proteins through protein refolding or by degrading them, thus restoring protein homeostasis and promoting cell survival [1]. They are a highly specialized and conserved group of proteins, which protect other proteins from misfolding and aggregation and allow them to fold efficiently on a cellular relevant timescale [2]. Metabolic stress including endoplasmic reticulum (ER) stress leads to accumulation and aggregation of unfolded proteins within the ER lumen. It is a central feature of peripheral IR and T2DM and has been implicated in diverse pathophysiological conditions such as cardiovascular diseases, cancer, aging, and obesity [3]. HSPs have been implicated in ER stress by activating unfolded protein response (UPR) including ER associated degradation of unfolded proteins [4, 5].

DNAJB3 is a JDP (J-domain protein) belongs to HSPs of the DNAJ/HSP40 family [6] which were found to be downregulated in peripheral blood mononuclear cells (PBMC) of obese patients [7]. The downregulation of DNAJB3 was associated with the activation of ER stress and this chaperone was part of a complex containing HSP72 along with JNK and IKKβ stress kinases [8]. Moreover, overexpression of DNAJB3 improves insulin signalling and glucose uptake in pre-adipocytes, suggesting DNAJB3 may have a protective role in obesity [9]. The overexpression of DNAJB3 was also linked to the activation of IRS-1 and subsequently AKT with AS160 phosphorylation—a substrate of AKT, suggesting DNAJB3 may also have a role in improving glucose uptake [9]. DNAJB3 was involved in promoting both basal as well as insulin-stimulated glucose uptake and participated in eliciting GLUT4 translocation to the plasma membrane [10]. Interestingly, silencing the expression of DNAJB3 abolished the protective effect of α-lipoic acid (ALA, an antioxidant compound), which was found to attenuate metabolic stress and improve insulin sensitivity induced by the ER stress activator, tunicamycin. This suggests, that DNAJB3 is a key mediator of ALA-alleviated tunicamycin-induced ER stress [11].

AKT1 is one of 3 closely related serine/threonine-protein kinases (AKT1, AKT2 and AKT3), regulating several cellular processes including metabolism, proliferation, cell survival, growth and angiogenesis [12–15]. These kinases work by phosphorylating a range of downstream substrates. Phosphorylation of AS160 (Akt Substrate Of 160 kDa), also known as TBC1D4 (TBC1 Domain Family Member 4), triggers the binding of this protein to inhibitory 14-3-3 proteins, which is required for insulin-stimulated glucose transport [16]. In terms of structural organization, AKT1 contains a PH domain (Pleckstrin homology domain) at N-terminal, followed by protein kinase domain and AGC-kinase C-terminal domain. Three specific sites, including one present in the kinase domain (Thr-308) and another two present in the C-terminal regulatory region (Ser-473 and Tyr-474), are phosphorylated to achieve full activation [17, 18]. Activation of AKT is triggered by the interaction between the lipid products of phosphoinositide 3-kinase (PI3K) and the PH domain of AKT. A conformational change is induced in AKT which exposes the activation segment of the kinase domain [19]. Upon insulin stimulation, AKT regulates glucose uptake in muscle, adipocytes and liver by phosphorylating and thus inactivating AS160 and thereby, promoting glucose transporters from intracellular stores to the plasma membrane with the concomitant increase in insulin-dependent transport of glucose into the cell [20, 21]. Given the importance of the biological processes regulated by these enzymes and the diversity of their downstream targets, the AKT pathway has been recognized as a promising therapeutic strategy for the treatment of various diseases linked to IR [22–24].

Here, we initially implemented a pull down technique to detect new binding partners of DNAJB3. The pull down assay revealed that AKT1 is an interacting partner of DNAJB3. Isothermal titration calorimetry (ITC) subsequently confirmed direct contact between these two proteins and quantified the avidity of AKT1 for DNAJB3. The binding interface was identified by combining protein modelling with docking of the complex and suggested a model by which the DNAJB3:AKT1 interaction specificity is achieved. However, the functional consequence of this interaction is unknown. DNAJB3 is localized in the cytoplasm and ER, where it interacts directly with AKT1 and mobilizes AS160 for glucose transport. Inhibition of AKT1 resulted in loss of GLUT4 translocation activity mediated by DNAJB3 and also abolished the protective effect of DNAJB3 on tunicamycin-induced ER stress. Taken together, our findings provide evidence for a direct protein-protein interaction between DNAJB3 and AKT1 upon which DNAJB3 alleviates ER stress and promotes GLUT4 translocation.

## 2. Materials and methods

### 2.1. Reagents

Anti-DNAJB3 antibody was purchased from Proteintech (Proteintech Group, Inc., Chicago, IL). Anti-AKT antibody was purchased from Cell Signaling (Cell Signaling Technology, Inc., Danvers, MA). Horse radish conjugated anti-GST antibody was purchased from Abcam (Abcam, Cambridge, UK). Insulin, wortmannin, ly294002, protease inhibitor cocktail, imidazole and reduced glutathione were purchased from Sigma (Sigma-Aldrich, St. Louis, MO). C2C12, HepG2 and 3T3-L1 were purchased from ATCC (ATCC, Manassas, VA). E. coli strains 10β and BL21 (DE3) were purchased from New England Biolabs (New England Biolabs, Ipswich, MA, USA). Glutathione resin was purchased from Pierce (Pierce, Rockford, IL, USA). Nickel-NTA resin, DNaseI and RNase A were purchased from Qiagen (Qiagen, Inc., Valencia, CA). Fluorescently labeled D-glucose analog (2-NBDG) was purchased from Cayman (Cayman, Ann Arbor, MI). Scrambled and specific siRNA were purchased from Dharmacon (Dharmacon Inc., Lafayette, CO). Lipofectamine 3000 and lipofectamine RNAiMAX were purchased from Invitrogen (Invitrogen, Carlsbad, CA). PureLinkTM RNA Minikit and High-Capacity cDNA Reverse Transcription Kit were purchased from Invitrogen (Invitrogen, Carlsbad, CA).

### 2.2. Plasmid constructs, primers and silencing RNA (siRNA)

The expression vector encoding human DNAJB3 (pCMV-DNAJB3; #RC205526), AKT (pCMV-AKT1; #R220257) and the pCMV empty vector (#pCMV6KN) were purchased from OriGene (OriGene Technologies, Inc, Rockville, MD). Plasmids encoding hDNAJB3 as a C-terminal fusion with GST (pGK-DNAJB3) and 6xHIS residues (pH6K-DNAJB3) were made by digesting pCMV-DNAJB3 with BglII/XhoI restriction enzymes and the insert was cloned into the unique BamHI/XhoI sites of pGK and the BamHI/SalI sites of pH6K, respectively. The same strategy was used to generate pGK-AKT and pH6K-AKT1 from pCMV-AKT1. The sequence integrity was verified by Sanger sequencing and the plasmids were used to transform *E*. *coli* strain B21 (DE3). Plasmid encoding GLUT4 as a fusion with HA epitope at the N-terminal and a GFP tag at the C-terminal (pHA-GLUT4-GFP) has been described previously [10]. The sequences of the siRNA used in this study are listed in Table 1.

**Table 1:** Primer list and sequences.

**Gene**	**Forward**	**Reverse**
DNAJB3	5′-AGGGGCTGTACCCTTCTCTA-3′	5′-AGTTTCCTGGAGAACCGAAG-3′
AKT1	5′-CTGCCCTTCTACAACCAGGA-3`	5′-CATACACATCCTGCCACACG-3’
AKT2	5′-CTTCGGCAAGGTCATTCTGG-3’	5′-TTGAGGGCTGTAAGGAAGGG-3’
ATF4	5′-GGGTTCTGTCTTCCACTCCA-3′	5′-AAGCAGCAGAGTCAGGCTTTC-3′
GRP78	5′-AATTTCTGCCATGGTTCTCA-3′	5′-AGCATCTTTGGTTGCTTGTC-3′
XBP1	5′-TCCCCAGAACATCTTCCCAT-3′	5′-ACATGACAGGGTCCAACTTG-3′
sXPB1	5′-CTGAGTCCGAATCAGGTGCAG-3′	5′-GTCCATGGGAAGATGTTCTGG-3′
GAPDH	5′-CTGGAGAAACCTGCCAAGTA-3′	5′-AGTGGGAGTTGCTGTTGAAG-3′
Actin	5′-AAGAGCTATGAGCTGCCTGA-3′	5′- GATGCCACAGGATTCCATAC-3′

### 2.3. Expression and purification of recombinant proteins from *E*. *coli*

Clones harboring DNAJB3 and AKT1, were inoculated in LB containing 100 mg/L ampicillin and incubated for overnight at 37°C using the shaker incubator. The next day, the starter culture was diluted 50 times in 2xYT media containing ampicillin and further cultured for approximately 2-3h at 37°C until the OD600 reached 0.6 and then, induced with 1 mM Isopropyl β-D-1-thiogalactopyranoside (IPTG) for 3h at 37°C. The bacteria pellet was obtained by a 10-min centrifugation at 10,000 rpm and then resuspended in HNG buffer-I (20 mM Hepes pH 8.0, 150 mM NaCl and 10% Glycerol). The remaining steps for protein purification using the Glutathione resin (for GST fusions) or Ni-NTA resin (for HIS fusions) were carried out as we described previously [25, 26]. To minimize non-specific binding of bacterial proteins to the resins, the binding was carried out in the presence of 1 mM reduced GSH (for GST resin) or 10 mM Imidazole (for Ni_NTA resin). After binding, the resins were washed twice with 10 times the bed volume of the resins with HNG Buffer-I, followed by another wash with HNG buffer–III (20 mM Hepes pH 8.0, 500 mM NaCl and 10% Glycerol) and a last wash with HNG buffer–IV (20 mM Hepes pH 8.0, 1 M NaCl and 10% Glycerol) and then, proteins were eluted in HNG buffer-I containing either 20 mM reduced GSH or 200 mM Imidazole. GST and Sox2-HMG proteins were made from pGK and pET-28 (a)-Sox2-HMG plasmids, respectively and used as negative controls for pull down experiments (GST) and isothermal titration calorimetry (Sox2-HMG). Proteins used for GST pulldown experiments were extensively dialyzed against HNG buffer-I (20 mM Hepes, 150 mM NaCl and 10% Glycerol). Proteins used for isothermal titration calorimetry were further separated by gel filtration chromatography. The affinity purified fractions containing the DNAJB3 and AKT were loaded in a HiLoad 16/600 Superdex 200 Peak fractions were collected and concentrated. Protein purity and integrity were monitored by Coomassie blue staining after separation on SDS-PAGE and the concentration was determined by Bradford assay using γ-globulin as a standard (BioRad, Hercules, CA, USA). Samples were aliquoted, snap frozen in liquid nitrogen and stored at -80°C.

### 2.4. Preparation of whole cell lysate

C2C12, 3T3-L1 and HepG2 cells (4x150 mm petri dishes for each cell type) were freshly propagated in DMEM media and allowed to grow until they reached a confluence of 80% and then collected for the preparation of whole cell lysates. After two washes with cold PBS, cells were trypsinized, and detached cells were centrifuged and the pellet was resuspended in 0.5 ml HNG buffer-I supplemented with 1 mM each of dithiotheritol (DTT) and phenylmethylsulfonyl fluoride (PMSF), protease inhibitor cocktail (PIC) and 20 μg/ml each of RNase A and DNase I, and then lysed by 3 cycles of sonication (30s per cycle). The cell suspension was made up to 500 mM NaCl, 1 mM EDTA and 1% (vol/vol) Triton X-100 and was mixed for 30 min at 4°C. Cell debris was removed by centrifugation for 1 h at 15,000 rpm and the supernatant was dialyzed overnight at 4°C against HNG buffer-I supplemented with 1 mM each of DTT, EDTA and PMSF and PIC. After 15 min centrifugation at 15,000 rpm at 4°C, protein concentration was determined by Bradford method and the samples were aliquoted, snap frozen and stored -80°C.

### 2.5. GST Protein pull-down experiments and Western blots

GST-tagged DNAJB3 protein (20 μg) and GST control protein (10 μg) were first incubated with 200 μl of a 50% slurry Glutatahione agarose resin, pre-equilibrated with cold HNG buffer-I and allowed to bind for 30 min at 4°C with constant rotation. Afterwards, 4 mg of whole cell lysates were added to the resin to give a final concentration of 8 mg/ml in 500 μl of total volume in the presence of 0.5% Tween and incubated at 4°C for 1h with constant rotation. To reduce non-specific binding of the cell lysates to the resin, they were pre-incubated first with resin alone for 30 min at 4°C with end-over end rotation, separated from the resin by centrifugation and then, applied to the resin containing GST and GST-tagged DNAJB3 protein. At the end of the incubation, the flow through was separated from the resin by centrifugation and the resin was subjected to 4 consecutive washes (1 ml each wash) with HNG buffer containing increased NaCl concentration (150 mM, 300 mM, 500 mM and 1M). Bound proteins were first eluted with 20 mM GSH. To recover the maximum of proteins, a second elution with 1% SDS was carried out. The 500 mM and 1M NaCl washes as well as the eluates were precipitated with 10% Trichloroacetic acid (TCA), washed in 100% Acetone and resuspended in 50 μl of 1x SDS-loading buffer. The enriched proteins were separated by a 12% SDS-PAGE gel and either stained with silver nitrate or transferred to nytran membrane for western blots using anti-AKT and GST antibodies.

### 2.6. Isothermal titration calorimetry (ITC)

The binding of DNAJB3 to AKT1 was carried out by ITC using a MicroCal Auto-iTC_200_ microcalorimeter (Malvern, USA) at 25°C. Before starting the measurements, both the proteins were purified by affinity and gel filtration chromatography, buffer exchanged in 25mM Tris-HCl pH 8.0, 150mM NaCl, 1mM dithiothreitol (DTT). The DNAJB3 (250 μM, syringe) was titrated into the sample cell, containing 20 μM Akt1, sequentially by injecting a 2 μL aliquot at each titration point with a time interval of 120s. Total of 19 injections of the DNAJB3 was mixed into AKT1 cell with stirring speed of 750 rpm. First initial small injection was used to minimize the impact of equilibration artifacts and was disregarded during evaluation of the data. Similarly, DNAJB3 (250 μM, syringe) was titrated into the sample cell, containing 20 μM Sox2-HMG protein. As a control experiment, buffer solution was used in sample cell with same concentration of DNAJB3 (250 μM) in syringe. All the sample were added in 96-deep well microplate before inserting the plate into tray. The heats of mixing and dilution of control experiment was subtracted from the heat of binding per injection. To determine the equilibrium dissociation constant (Kd) and the enthalpic change (ΔH) associated with binding, the ITC isotherms were iteratively fit to a one-site binding model by non-linear least squares regression analysis using the integrated ORIGIN software.

### 2.7. Protein modelling and Docking (DNAJB3-AKT1 interaction)

The partial structure of DNAJB3, J-domain structure is known (PDBID: 2EJ7), to reconstruct the full length DNAJB3, we generated its structure model by Swiss-Model [27]. Amino acids sequence of the human DNAJB3 (hDNAJB3) was submitted. Based on the protein sequence coverage and sequence identity, NMR structure of the DNAJB6b (PDBID: 6U3R) was selected for homology modelling. Three-dimensional structural models of hDNAJB3 was generated and the quality of the predicted model was examined by MolProbity [28], a structure validation server, which revealed the results in terms of phi, psi and Cβ deviations by generating a Ramachandran plot for the resulted model. The quaternary structure analysis was performed by QSQE—a tool for the prediction of correct quaternary structure by combination of conservation scores, structural clustering, and classical interface descriptors [29]. Similarly, we also generated three-dimensional structural models of mouse DNAJB3 (mDNAJB3) by the Swiss-model server using the NMR structure of the DNAJB6b (PDBID: 6U3R) as the search model.

We performed molecular docking using HDOCK docking server [30, 31]. For docking studies, AKT1 (PDBID:3CQW, comprising 144–480 aa) were retrieved and manually processed to remove water and heteroatoms. As AKT1 was bigger in size, it was used as a receptor and modelled DNAJB3 as ligand. We performed the docking with default parameters and 10 predicted conformers (poses) were further analysed. All structural analysis, figures with structure representations and superimposition were produced using the program UCSF Chimera [32].

### 2.8. DNAJB3 sub-cellular localization with confocal microscopy

To examine DNAJB3 sub-cellular localization, C2C12 cells were grown on 12-well plates containing coverslip until they reached 80% confluency and then transfected with 7.5 μg DNAJB3 or DNAJB3 tagged with GFP (DNAJB3-GFP) plasmids using Lipofectamine 3000 (Invitrogen, Carlsbad, CA). 24h following the transfection, cells were fixed with 4%PFA, permeabilized with 0.1% Triton X-100 and blocked with 3% BSA for 60 minutes at room temperature. Cells transfected with DNAJB3 plasmid were incubated with antibody against DNAJB3 overnight while those transfected with DNAJB3-GFP were incubated with antibody against GRP78 (an ER marker). Cy3-conjugated Goat Anti-Rabbit was used as the secondary antibody. DNAJB3 (GFP) and GRP78 (stained in red) and DAPI (blue) was used as control for nuclear staining. Images were acquired using a 25×/0.8 numerical aperture (NA) objective (LD LCI Plan-Apochromat; Carl Zeiss Inc., Oberkochen, Germany) using confocal microscopy on a laser scanning microscope (LSM 780; Carl Zeiss Inc., Oberkochen, Germany), mounted on a laser scanning microscope (LSM) (Zeiss LSM 780; Carl Zeiss Inc.,). Images were analyzed using ZEN imaging software (Carl Zeiss Inc.).

### 2.9. Transient transfections to silence AKT expression using siRNA

C2C12 were grown on 6-well plates at 200,000 cells/well in an antibiotic free DMEM media supplemented with 10% FBS at 37°C and 5% CO2 until they reached 80% confluency and then transfected with 20 nM of smart pool Accell siRNA targeting mouse AKT (AKT-siRNA) or Accell non-targeting control (scrambled-siRNA; D-001910-10-05, Dharmacon Inc., Lafayette, CO) to knock-down the expression of AKT using lipofectamine RNAiMAX from Invitrogen (Invitrogen, Carlsbad, CA) under a serum free media conditions. After 48h of transfection, ER stress was induced with tunicamycin at a dose of 0.5 μg/ml overnight afterwards, cells were harvested for RNA extraction. All the assays were performed at least in triplicate and in three independent experiments.

### 2.10. Measurement of gene expression by real-time RT-PCR

RNA was extracted from treated cells using PureLink^TM^ RNA Minikit as instructed by the manufacturer. It was then converted to cDNA using High-Capacity cDNA Reverse Transcription Kit and analyzed by RT-PCR on QuantStudio 6 Flex system using SYBR Green (ThermoFisher, Waltham, MA). Relative expression was calculated by the Livak comparative ΔΔCt method [33]. Briefly, relative gene expression was calculated by the difference (ΔCt) between the Ct value of the gene of interest and that of the reference gene. Then the difference in the delta value between the experimental and control group (ΔΔCt) and the fold change (2^-ΔΔCt^) were calculated. GAPDH and actin genes were used as internal controls. The primers corresponding to genes of the ER stress have been published previously [11]. The sequences of the primers are listed in Table 1.

### 2.11. GLUT4 translocation monitoring by flow cytometry

C2C12 were grown on 6-well plates at 200,000 cells/well in an antibiotic free DMEM media supplemented with 10% FBS at 37°C and 5% CO2 until they reached 80% confluency and then transfected with 3.75 μg of HA-GLUT4-GFP plasmid and 3.75 μg of either DNAJB3 or pCMV using Lipofectamine 3000 (Invitrogen, Carlsbad, CA). The following day, half of the wells with cells transfected with both HA-GLUT4-GFP and DNAJB3 plasmids were treated overnight with either LY294002 (40 uM) or Wortmannin (100nM), pharmacological inhibitors of phosphoinositide 3-kinases (PI3K), an upstream kinase involved in AKT signalling pathway. After glucose and serum starvation for 1h, cells were stimulated with 100 nM of Insulin (Sigma Aldrich, St. Louis, MO), trypsinized, collected in falcon tubes (100,000 cells/tube) and subjected to HA staining using mouse Anti-HA Tag Alexa Fluor® 647-conjugated Monoclonal Antibody (Catalog # IC6875R) for 30 min. Thereafter, cells were washed 2 times with FBS stain buffer and the HA Tag Alexa Fluor 595 (labelling only the GLUT4 on the plasma membrane) and GFP (reflecting the total Glu4 expression) positive cells were determined by flow cytometry. The surface-to-total GLUT4 ratio (HA/GFP) was calculated to determine the GLUT4 translocation as previously reported [11].

### 2.12. Statistical analysis

All assays were performed at least in triplicate and a minimum of three independent experiments. Results are presented as means ± SEM and were plotted using GraphPad (Prism v7, La Jolla, CA). Shapiro-Wilk test was first performed for the normality test followed by parametric tests. We used unpaired t-tests or two-way analysis of variance to test gene (Scrambled- vs SiRNA) by treatment condition (vehicle vs tunicamycin) effects and one-way analysis of variance for comparison of the groups with post-hoc Tukey’s test for pairwise comparisons. A P-value <0.05 was considered statistically significant.

## 3. Results

### 3.1. Identification of AKT1 as an interacting partner of DNAJB3

**Fig 1:**
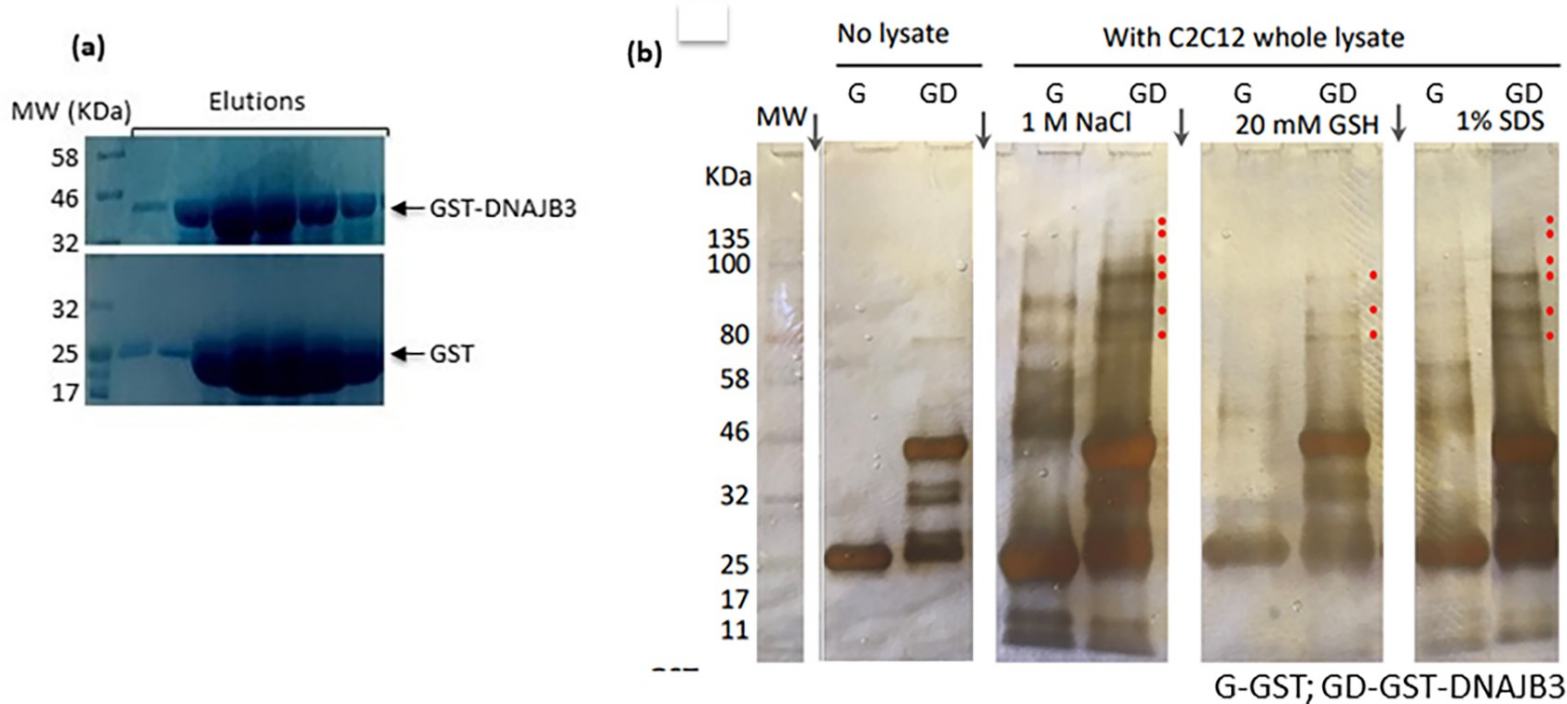
AKT1 interacts specifically with DNAJB3. (a) SDS-PAGE analysis of the recombinant GST-DNAJB3 and GST control proteins expressed and purified from E. coli. Different fractions of the elutes were resolved by SDS-PAGE and stained with Coomassie blue. (b) GST-pull down experiments were carried out using GST-DNAJB3 recombinant protein or GST (a negative control) and whole cell lysate prepared from C2C12 cells. After binding, the resin was washed extensively with the binding buffer containing increasing concentrations (0.1–1 M) of NaCl. Bound proteins were eluted with 20 mM GSH followed by 1% SDS, resolved by SDS-PAGE and stained with silver nitrate. Proteins present in the 1 M NaCl wash were loaded in parallel. The binding of GST and GST-DNAJB3 without lysate was also included. The protein bands that interact specifically with GST-DNAJB3 are indicated by a red dot. As similar pattern was also obeseved in 3T3-L1 adipocytes and HepG2 cells (S1 Fig in S1 File). Splice points are shown as vertical arrows.

In our previous study, we found that DNAJB3 overexpression resulted in increased phosphorylation of AKT1 enzyme and its substrate AS160 as well as enhanced glucose uptake in 3T3-L1 adipocytes [9]. Furthermore, we reported recently a role of DNAJB3 on stimulating GLUT1 mRNA expression and GLUT4 protein translocation to the plasma membrane in the skeletal muscle C2C12 cells [10]. In this study, we examined the possible interaction between DNAJB3 and AKT enzyme. To this end, DNAJB3 was expressed and purified to near homogeneity from *E*.*coli* as a GST-fusion protein (Fig 1A), and used in pull down experiments using glutathione agarose resin. GST protein was purified in parallel with DNAJB3 and used as a negative control (Fig 1A). Both DNAJB3 and GST have been dialyzed extensively and used as column ligands to capture possible partners present in the whole cell lysates prepared from C2C12 cells as indicated in materials and methods. After extensive washes (up to 1 NaCl), bound proteins were eluted with 20 mM reduced glutathione (GSH) followed by 1% sodium dodecyl sulfate (SDS), resolved by SDS-polyacrylamide gel electrophoresis (PAGE), and stained with silver nitrate (Fig 1B). Accordingly, several polypeptides with apparent masses ranging from 60 kDa and 180 KDa were detected in the 1M NaCl wash and the elutions of the DNAJB3 bound resin found to be selective binders of DNAJB3 (Fig 1B, lanes 4, 6 and 8; splice points in gel are marked with vertical arrows) and not to the GST (Fig 1B, lanes 3, 5 and 7). As a further control, no such polypeptides were present in the 1% SDS eluate from resins that had not received C2C12 lysate (Fig 1B, lanes 1 and 2). Hence, the recovery of these polypeptides was dependent on both DNAJB3 and C2C12 lysate (Fig 1B). To investigate whether the observed pattern was specific to the skeletal muscle C2C12 cells or similar to other metabolically relevant cells such as 3T3-L1 adipocytes and HepG2 liver cells. As displayed in Supplementary figure (S1 Fig in S1 File), a consistent pattern was observed in the 1% SDS elutes in all the cells.

To investigate whether AKT1 was among the polypeptides found in DNAJB3 bound resin, the 1M NaCl wash and the elutes were resolved on SDS-page, transferred to a membrane and screened against anti-AKT antibody. In parallel, anti-GST was used as internal control. Results revealed the presence of AKT1 protein in DNAJB3 but not in GST (S1 Fig in S1 File).

### 3.2. Validation of DNAJB3: AKT1 interaction and determination of binding parameters

**Fig 2:**
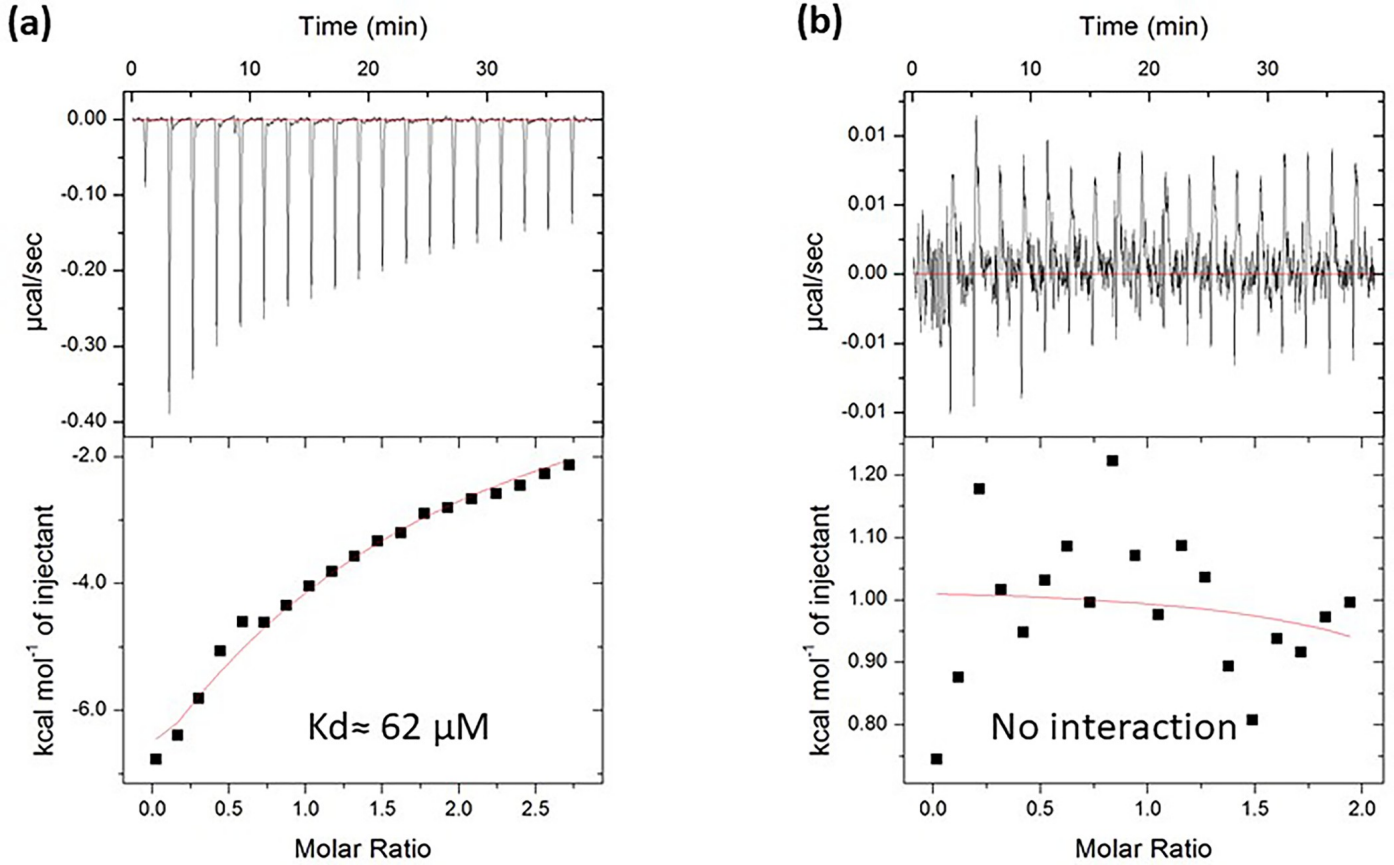
ITC analysis of DNAJB3: AKT1 interaction. (a) ITC titration curve (upper panel) and binding isotherms (low panel) of DNAJB3: AKT1 interactions. The upper panels show the raw ITC data expressed as change in thermal power with respect to time over the period of titration. In the lower panels, change in molar heat is expressed as a function of molar ratio of titrant. The solid line in the lower panels indicates the non-linear least squares fit for the integrated data to a one-site binding model using the integrated ORIGIN software. The solid line in the lower panels indicates the non-linear least squares fit for the integrated data to a one-site binding model using the integrated ORIGIN software. (b) ITC measurements showing of titration curve (upper panel) and binding isotherm of DNAJB3 titration into Sox2-HMG protein. No binding was observed.

To directly provide the evidence of DNAJB3: AKT1 interaction, we measured the binding of DNAJB3 to AKT1 using isothermal titration calorimetry (ITC). Both the proteins were purified recombinantly (His-DNAJB3 and His-AKT1) from *E*.*coli* with similar buffer composition (S1 Fig in S1 File). Both the proteins used for isothermal titration calorimetry (ITC) were His-tagged compared to GST-tagged DnajB3 used for Pull down. ITC requires a highly homogenous and pure proteins, thus we employed gel filtration chromatography to get homogenous population of the proteins. The representative ITC isotherms for the binding of DNAJB3: AKT1 are shown in Fig 2. We found that DNAJB3 interacts with AKT1 with mid-range binding affinity of approximately 62 μM (Stoichiometry, N = 0.84), possibly a transient binding. In parallel, to eliminate the contribution of His-tag, we performed the ITC measurements of DNAJB3: His-Sox2 HMG (common protein involved in DNA binding) and analyzed the isotherm. We did not observe any binding under similar condition used for DNAJB3: AKT1 interaction. The binding of DNAJB3 to AKT1 was predominantly driven by favorable enthalpic forces (-ve value of ΔH) suggesting the formation of specific intermolecular interactions such as hydrogen bonding and van der Waals contacts that likely highlight the specificity of this protein-protein interaction.

### 3.3. Structural analysis of DNAJB3 and AKT1 and their interaction

**Fig 3:**
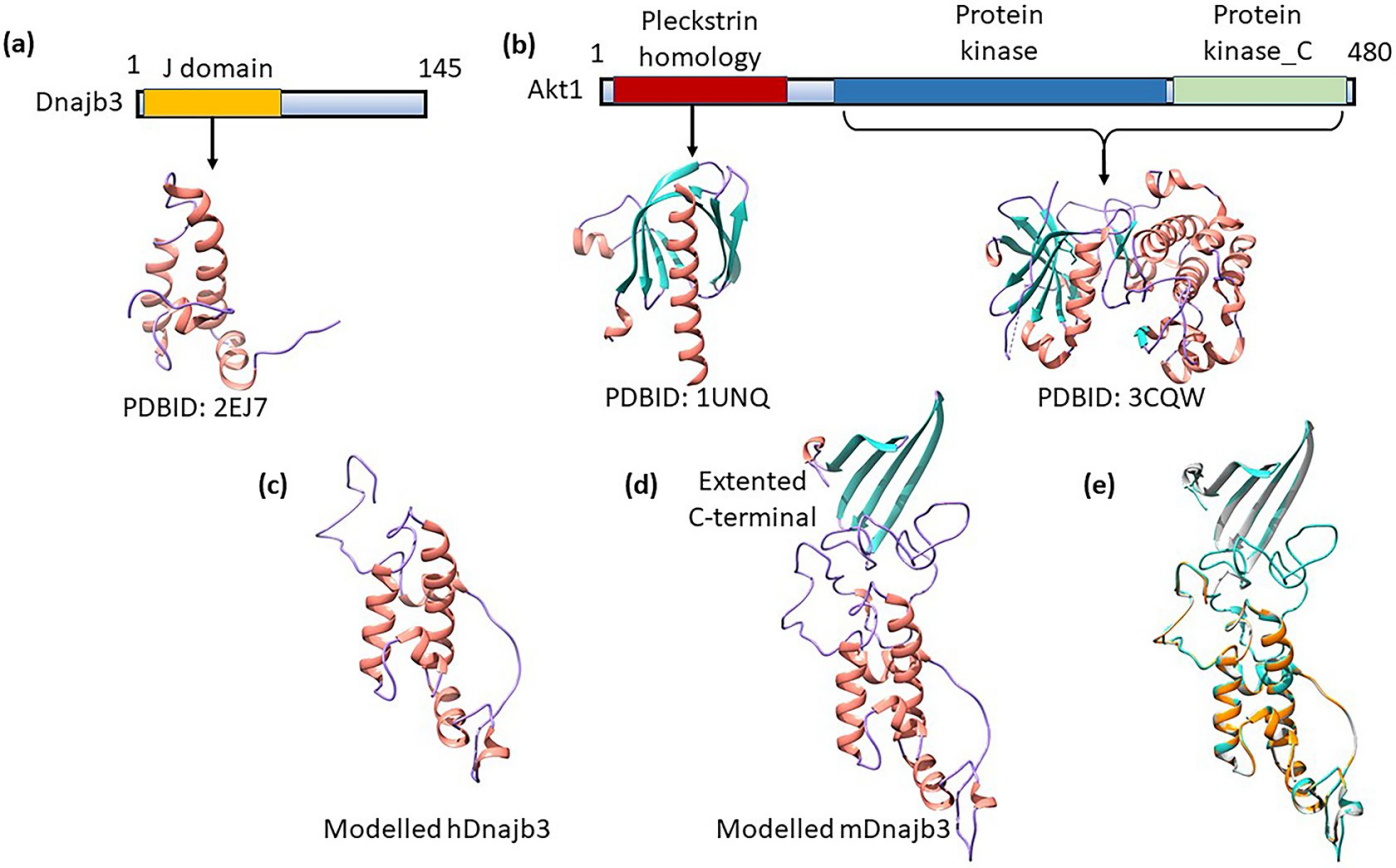
Structural details of DNAJB3 and AKT1. (a) Modular organization of human DNAJB3 protein. DNAJB3 contains the canonical J domain near N-terminal and a long C-terminal portion. While J domain structure is known, the C-terminal structure is unknow. (b) Modular organization of AKT1 protein. AKT1 is comprised of three well known domains, designated Pleckstrin homology (PH) domain located at N-terminal, Protein kinase domain in the middle and Protein kinase_C domain, located at C-terminal. (c) Cartoon representation of the overall modelled human DNAJB3 (hDNAJB3) structure. Secondary structures are shown in salmon for α-helix, and purple for loops. (d) Cartoon representation of the overall modelled mouse DNAJB3 (mDNAJB3) structure. Secondary structures are shown in salmon for α-helix, sea green for β-strands, and purple for loops. (e) Structural alignment of modelled hDnajb3 (orange), mDNAJB3 (sea green) and NMR structure of the DNAJB6b (PDBID: 6U3R, grey). The structures are highly similar in overall fold with protrusion of C-terminal as CTD in case of mDNAJB3.

To delineate the structural basis of interactions, we investigated the structural details of both the proteins. Human DNAJB3 is 145 amino acids (145 aa) protein with J-domain (1–69 aa) at the N-terminal (Fig 3A). J domain is a common conserved domain found in Hsp40s and interacts with Hsp70 to carried out chaperone function. AKT1 is a 480 aa, three domain protein: Pleckstrin homology (PH) domain (5–108 aa), located at N-terminal, Protein kinase domain (150–408 aa) in the middle and Protein kinase_C domain (409–480 aa), located at C-terminal (Fig 3B). PH domain is known to bind phosphatidyl inositol lipids and found in proteins involved in intracellular signalling. Protein kinase domain is the central domain within AKT1 and catalyses the phosphorylation and involved as an on/off switch in multitude of cellular processes. Protein Kinase_C (PKC) phosphorylates serine and threonine of several proteins and mainly involved in signal transduction cascade. Several structures of full length, individual domain and combination of domains are known (For example, PDBID: 1UNQ- PH domain; PDBID: 3CQW- Protein kinase+ PKC; PDBID: 4EJN- Full length).

To generate the full length DNAJB3 structure, we performed the homology modelling using SWISS-MODEL [27]: an automated server to generate 3D models of proteins. The protein sequence submitted retrieve potential templates for homology modelling. We selected the PDBID: 6U3R (DNAJB6b) for a template based on the highest global model quality estimation (GMQE) score of 0.77; quality estimation which combines properties from the target-template alignment. The modelled structure contains N-terminal region from residues 1 to 69 comprising of 4 α helices (Fig 3C), consistent with previously solved solution structures of J domains [34–36]. The C-terminal contains mainly loop regions. Structural superimposition between homology modelled DNAJB3 and Alphafold predicted structure of the DNAJB3 showed that the structures are similar, with a rmsd value of 1.07 Å for 62 Cα atoms. The difference lies in the unstructured regions of the protein. These regions have very low per-residue confidence score (pLDDT) in Alphafold prediction ((pLDDT < 50) suggesting that these regions have high flexibility and cannot be predicted with high accuracy. Interestingly, the close homolog from mouse contains an extensive C-terminal region of around 100 aa (S1 Fig in S1 File). Homology modelling of mDNAJB3 (Fig 3D) using 6U3R as template reveals J domain topology for N-terminal region and C -terminal adopt 4 β-strand similar to C-terminal substrate binding domain (CTD) of DNAJB6b [34]. The Structural comparison between modelled hDNAJB3 and mDNAJB3 (Fig 3E) shows root mean square deviations (rmsd) of 0.67 Å for 126 Cα atoms, indicating that the overall conformations of these two structure remain same upto residue 126, however the CTD domain is absent in hDNAJB3. Structural alignment by superimposition between modelled mDnajb3 and NMR structure of the DNAJB6b (PDBID: 6U3R) showed that the structures are similar, with a rmsd value of 1.15 Å for 189 Cα atoms (Fig 3E).

**Fig 4:**
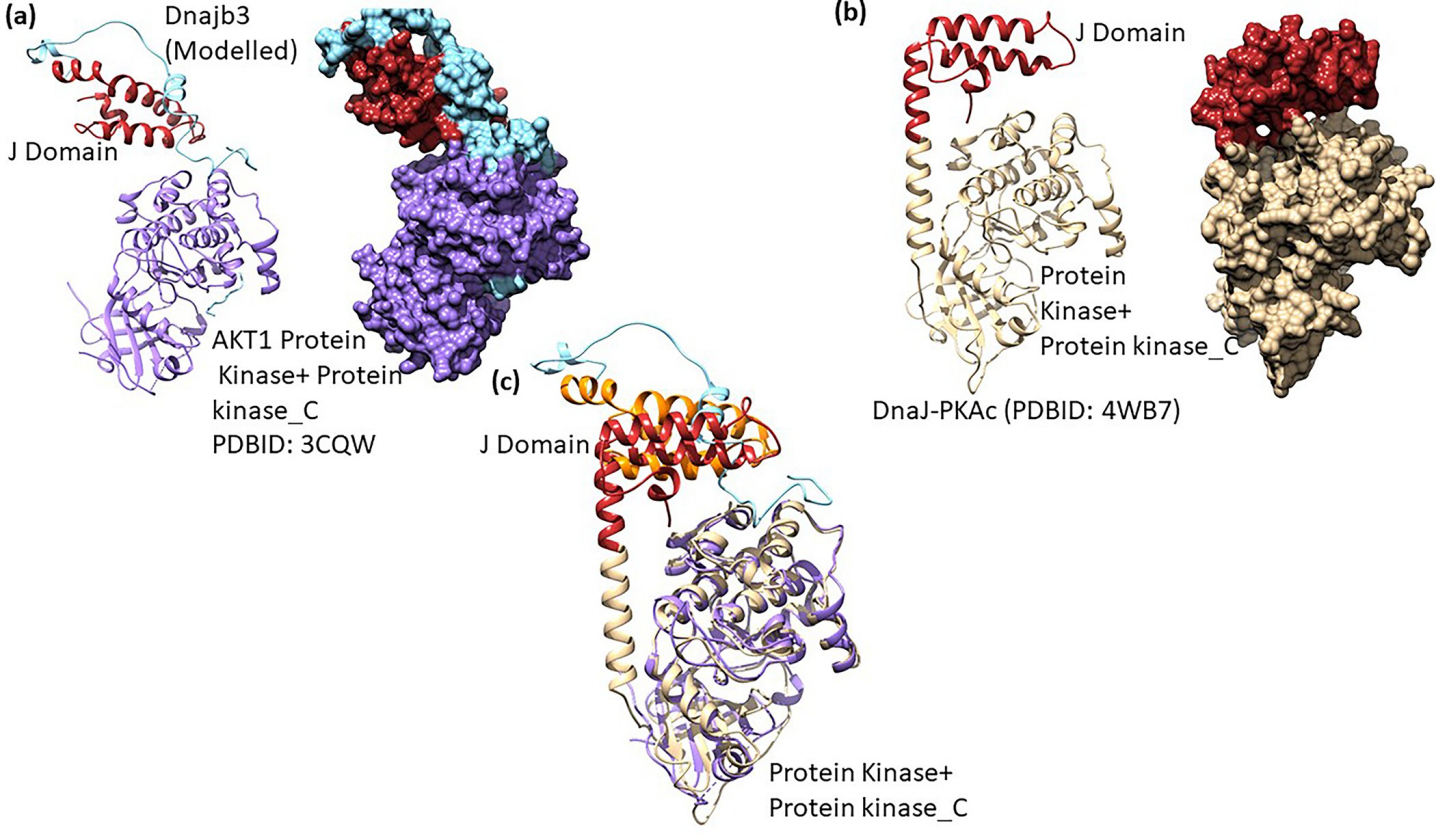
Docking of modelled DNAJB3 onto AKT1 and structural comparison. (a) Molecular docking of modelled DNAJB3 (J domain: red, c-terminal: sea green) onto AKT1 (PDBID:3CQW, purple) represented as cartoon (left panel) and surface (right panel). (b) Structure of Dnaj-PKACα (PDBID: 4WB7) drawn in carton (left panel) and surface (right panel) representations. The PKACα is coloured golden and J domain as red. (c) Structure alignment of docked (DNAJB3: AKT1) structure to Dnaj-PKACα (PDBID: 4WB7).

Molecular docking of modelled DNAJB3 and AKT1 was performed by HDOCK docking server [30, 31]. HDOCK is a fast protein–protein docking server automatically predicts their interaction through a hybrid algorithm of template-based and template-free docking [30]. The modelled DNAJB3 docked at the AKT surface created by the interface of two adjoining domains (Protein kinase and PKC) (Fig 4A). The interacting interface comprises residues from DNAJB3 (Lys25, Asn35, Glu37, Asn38) and formed charge based interaction with AKT residues (Glu247, Asp248, Arg243). A naturally occurring chimeric protein, DnaJ–PKAcα has the N-terminal J domain and C-terminal protein kinase and PKC domain [37, 38]. The structure of this chimeric protein (PDBID: 4WB7) highlights the conservation of relative orientation of the four helices J domain and is partially buried against the large conserved kinase core (Fig 4B) [38]. When the structures of docked DNAJB3:AKT1 and chimeric protein were superimposed (Fig 4C), it was clear that the J domain binds similarly in the vicinity of kinase region of the protein. However, in molecular dynamics simulations the fused J domain explores large conformational space and also rotates away from the kinase core [37, 39], suggesting that the docked or crystal structure is one of the conformation of the J domain relative to kinase core.

### 3.4. DNAJB3 sub-cellular localization

**Fig 5:**
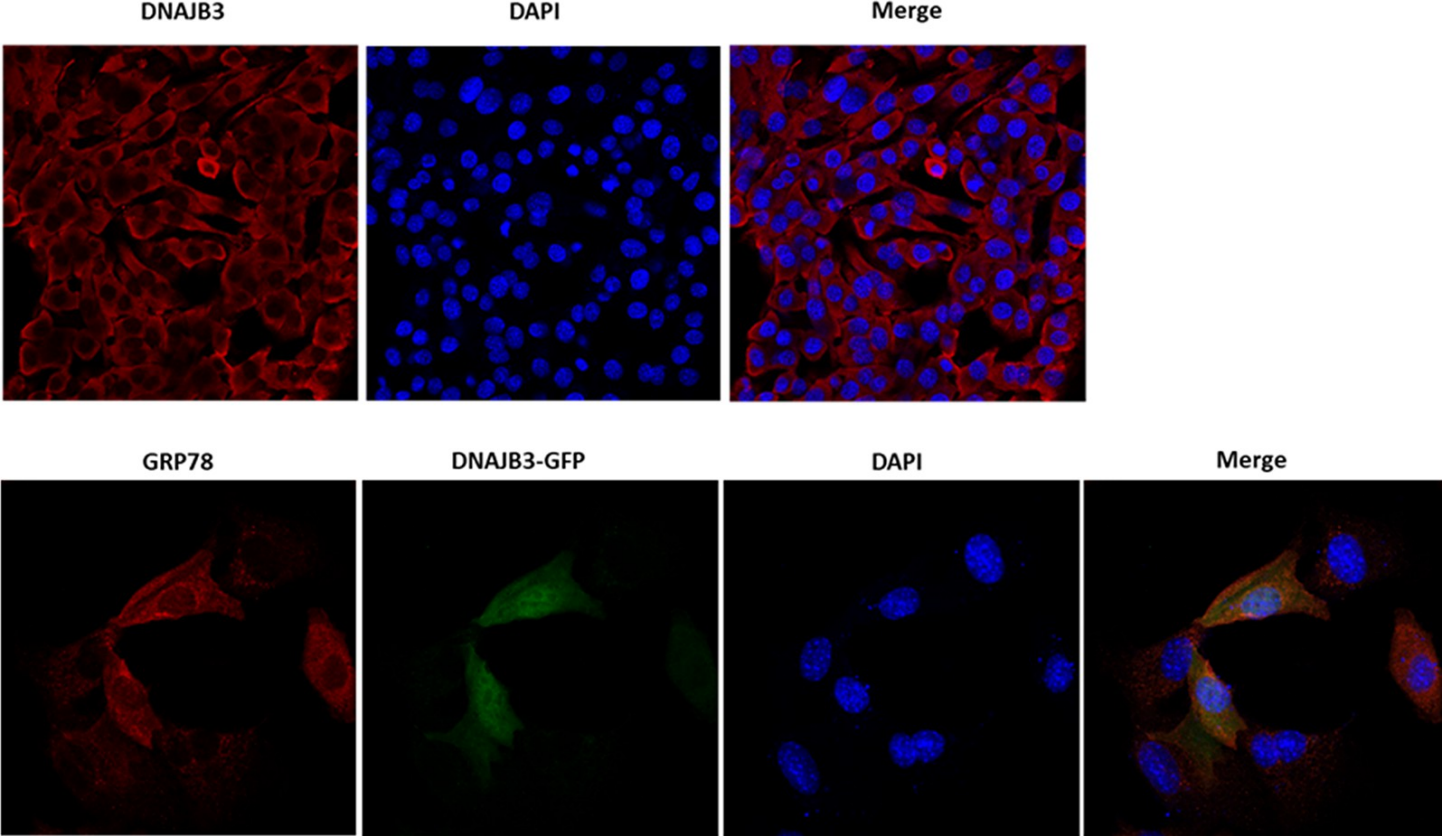
DNAJB3 sub-cellular localization with ER marker using confocal microscopy. Staining with anti-DNAJB3 in C2C12 cells reveals its non-nuclear localization (upper panel). C2C12 cells were transfected with DNAJB3 tagged with GFP (DNAJB3-GFP) and stained with anti-GRP78 (an ER marker) and found that they colocalize (lower panel). DAPI was used as control for nuclear staining.

Confocal images showed the presence of DNAJB3 in the cytoplasm and not in the nucleus; thus confirming the electron microscopy findings [40]. To further investigate whether DNAJB3 is localized in the ER, we stained DNAJB3-GFP transfected cells with anti-GRP78 (an ER marker). Representative confocal images after merging showed that DNAJB3 is present in both cytosol and ER but not in the nucleus (Fig 5).

### 3.5. Silencing the expression of AKT abolished the beneficial effect of DNAJB3 on alleviating ER stress

**Fig 6:**
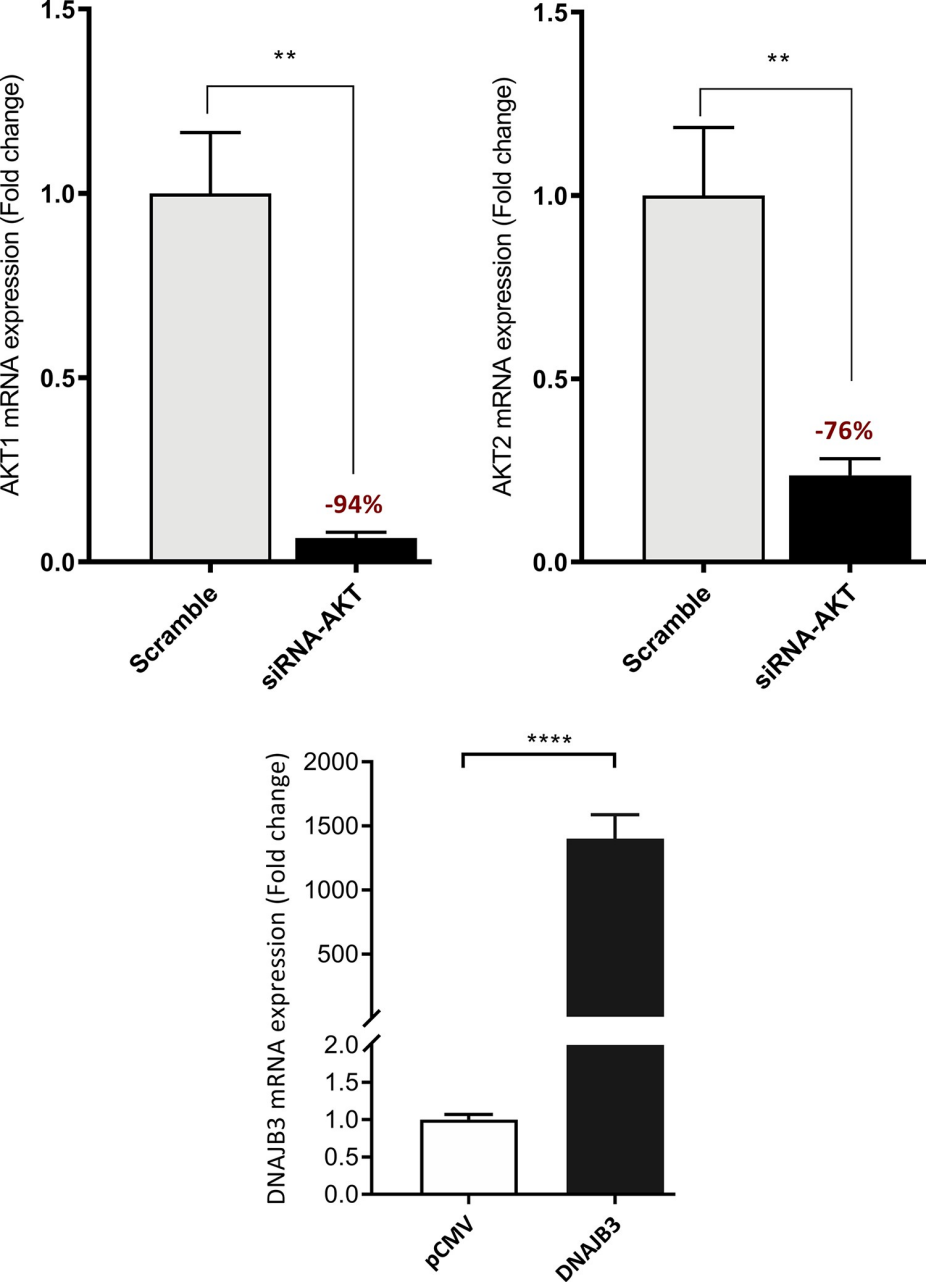
AKT Knockdown and DNAJB3 overexpression transfection efficiency in C2C12 cells. Knocking down the expression of AKT with 20 nM of specific siRNA blunted the endogenous expression of AKT mRNAs. Actin was used as internal control for RT-PCR (upper panel). Transient overexpression of DNAJB3 in C2C12 cells (lower panel). All assays were performed at least in triplicate and a minimum of three independent experiments. Results are presented as means ± SEM and were plotted using GraphPad (Prism v7, La Jolla, CA). Shapiro-Wilk test was first performed for the normality test followed by parametric tests.

**Fig 7:**
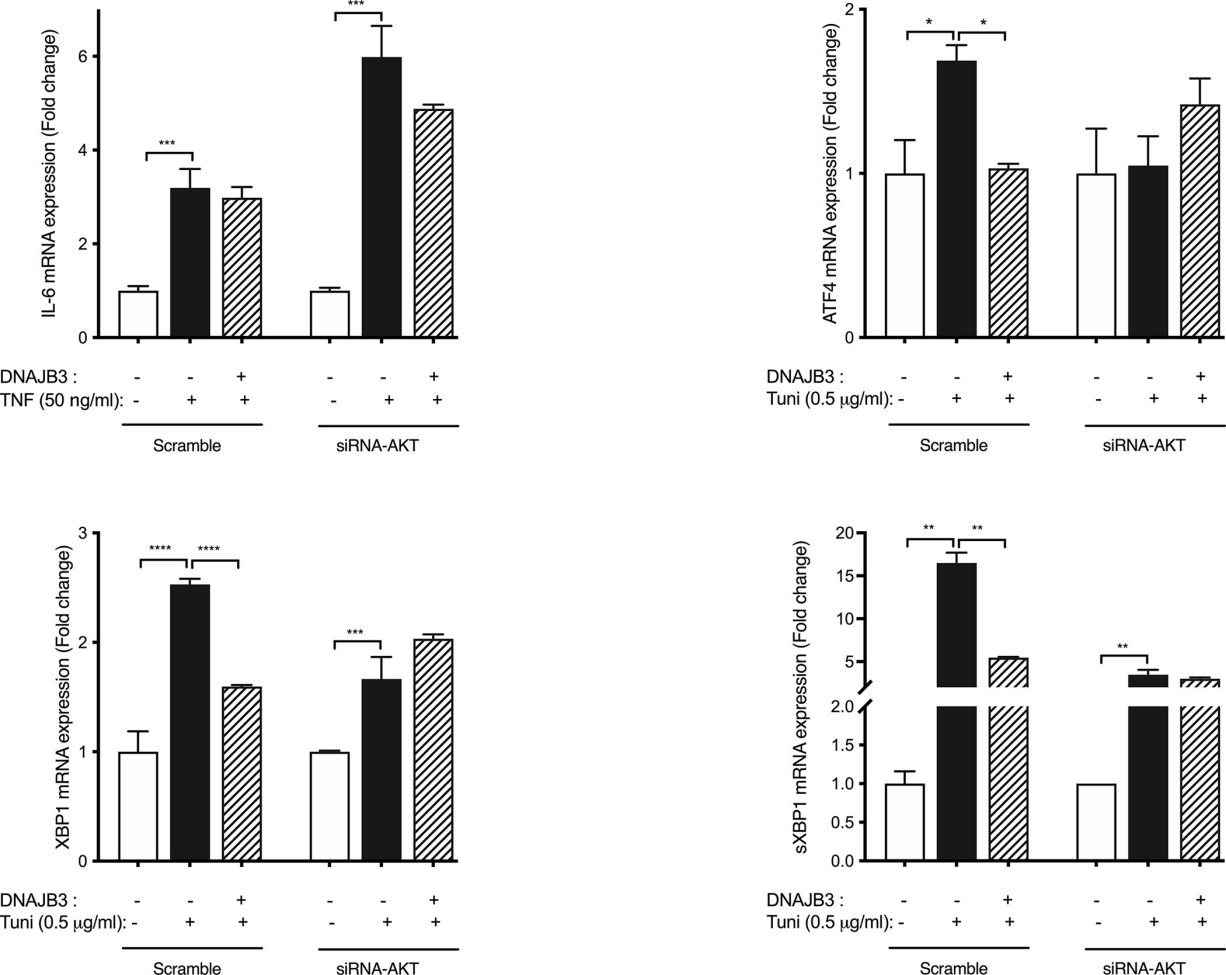
Relevance of AKT in DNAJB3-mediated ER stress. Silencing the expression of AKT abolishes the protective effect of DNAJB3 on tunicamycin-induced ER stress in C2C12 cells. DNAJB3 fails to protect siRNA AKT-transfected C2C12 cells from tunicamycin-induced mRNA expression of ER stress marker (GRP78). All assays were performed at least in triplicate and a minimum of three independent experiments. Results are presented as means ± SEM and were plotted using GraphPad (Prism v7, La Jolla, CA). Shapiro-Wilk test was first performed for the normality test followed by parametric tests. We used unpaired t-tests or two-way analysis of variance to test gene (Scrambled- vs SiRNA) by treatment condition (vehicle vs tunicamycin) effects and one-way analysis of variance for comparison of the groups with post-hoc Tukey’s test for pairwise comparisons. A P-value <0.05 was considered statistically significant.

We previously found that DNAJB-transfected cells showed decrease expression of endogenous genes of ER stress in response to tunicamycin [10]. To investigate the possible role of AKT in DNAJB3 alleviating ER stress, we silenced the expression of AKT using siRNA. As shown in Fig 6, 20 nM of siRNA-AKT blunted effectively the expression of both AKT1 and AKT2 mRNA in C2C12 myoblasts by 94% (P<0.0001) and 76% (P<0.0001), respectively. We then examined the effect of knocking down the expression of AKT on ER stress and as displayed in Fig 7, knocking down the expression of AKT with specific siRNA, abolished the beneficial effect of DNAJB3 on attenuating tunicamycin-induced ER stress marker genes, namely GRP78, ATF4, XBP1 and sXBP1, as compared to scrambled; this suggested AKT as a molecular determinant through which DNAJB3 mediates its beneficial effect on ER stress. In addition we also analyzed the effect of TNF-induced ER stress by monitoring IL-6 expression and note that there was a relatively subdued rescue effect relative to tunicamycin induced ER stress and furthermore AKT had minimal effect relative to DNAJB3 alone.

### 3.6. Pharmacological inhibition of AKT abolished the beneficial effect of DNAJB3 on insulin-promoted GLUT4 translocation

**Fig 8:**
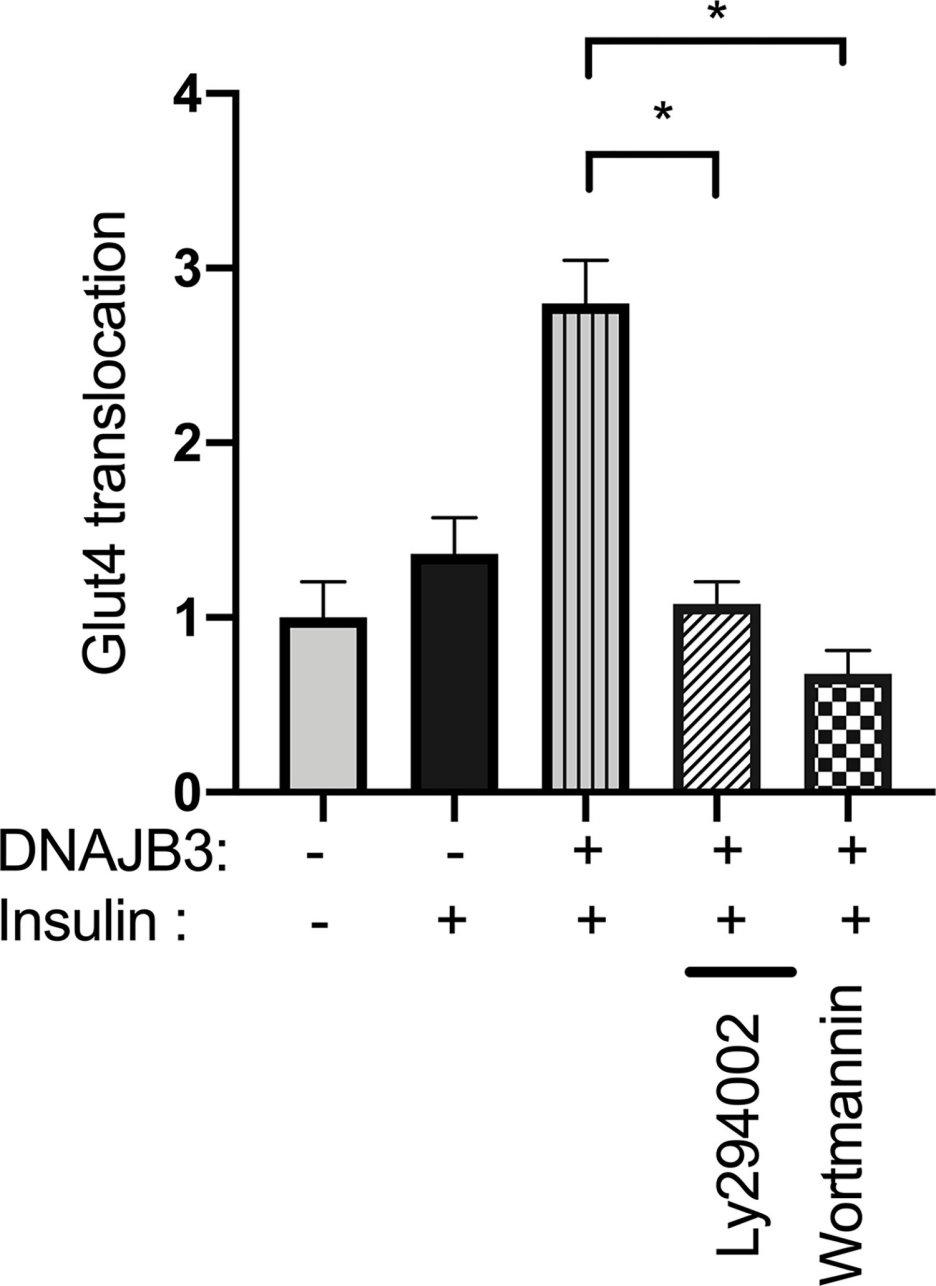
Relevance of AKT in DNAJB3-mediated GLUT4 translocation. Pharmacological inhibition of AKT with LY294002 (40 uM) or Wortmannin (100nM) abrogated significantly DNAJB3 effect on GLUT4 translocation to the plasma membrane in C2C12 cells. All assays were performed at least in triplicate and a minimum of three independent experiments. Results are presented as means ± SEM and were plotted using GraphPad (Prism v7, La Jolla, CA). Shapiro-Wilk test was first performed for the normality test followed by parametric tests.

GLUT4 transporters have a key role in insulin-mediated glucose uptake by the skeletal muscle. We previously demonstrated a marked increase in insulin-stimulated GLUT4 translocation in DNAJB3 transfected cells as compared to pCMV control [10]. To get more insight into a possible role of AKT, we inhibited its upstream regulator, PI3K, with LY294002 or Wortmannin. As expected, insulin-stimulated GLUT4 translocation is higher in cells overexpressing DNAJB3 as compared to pMCV, replicating our previous findings. However, the DNAJB3 promoting effect on insulin-stimulated GLUT4 translocation into the plasma membrane is blunted when AKT pathway is inhibited with either 40 uM of LY294002 or 100 nM Wortmannin (Fig 8).

## 4. Discussion

The coordinated interactions between molecular chaperones, kinases and other regulatory proteins with scaffolding properties is an emerging field in intracellular signal transduction activated by growth factors, cytokines and hormones [41]. A well-studied example is the insulin signaling pathway which involves several stimulatory and inhibitory serine/threonine kinases, phosphatases and other adaptor proteins [42, 43]. Thus, the impact of insulin stimulation on the biological response is dictated by the balance between the various kinases, phosphatases and other regulatory proteins. Recent studies demonstrated a convincing role of HSPs in potentiating the insulin response by counter regulating (interfering with or suppressing) the inhibitory kinases that abrogate the insulin action (that interfere with the insulin receptor-mediated signaling) such as JNK and IKK [10, 44, 45], while promoting the PI3K-AKT pathway, which is crucial for the metabolic action elicited by insulin in peripheral tissues [23, 46–48].

We previously demonstrated a role of DNAJB3 in stimulating the AKT1 pathway [9], attenuating ER stress and promoting GLUT4 translocation to the plasma membrane [10], however the molecular mechanisms underlying DNAJB3-mediated AKT1 activation has never been studied. Using a pull down approach combined with ITC, we provide in the present study evidence for a direct interaction between DNAJB3 and AKT1. The ability of DNAJB3 to attenuate tunicamycin-induced ER and to stimulate GLUT4 translocation in cell-based assays were significantly abrogated upon silencing the expression of AKT1 with siRNA or (and) blocking its activity with pharmacological drugs. DNAJB3 reduces the ER stress following TNF or tunicamycin stimulation, suggestive of a role in alleviating the ER stress. However, in absence of AKT, DNAJB3 fail to rescue the ER stress induced by tunicamycin as observed on the endogenous expression of representative markers of ER stress; namely XPB1 and ATF4. In mammalian cells, the unfolded protein response comprises at least three parallel intracellular signaling pathways controlled by ER-resident transmembrane proteins: inositol-requiring enyzme-1 (IRE1), PKR-like ER kinase (PERK), and activating transcription factor-6 (ATF6), by which cytoprotective mechanisms are initiated to restore ER homeostasis [49, 50]. XBP1 and s-XBP1 are a specific molecular readout for IRE1 activity while ATF4 is a molecular marker for monitoring PERK activity [50–52]. Contrary to XBP1 and s-XBP1, in the absence of DNAJB3 tunicamycin failed to trigger a higher expression of ATF4 following AKT silencing; suggesting that the PERK pathway is not required for DNAJB3 and AKT interaction to restore ER functions. These multiple regulatory mechanisms help in modulating the response to various levels of ER stress. Taken together these findings are the first to demonstrate a physical interaction between DNAJB3 and AKT1 and add further evidence to the importance of DNAJB3 in controlling diseases linked to IR. They also suggest that a functional AKT1 is required for DNAJB3 action in metabolic diseases.

DNAJB3 is a JDP and member of the DNAJ/HSP40 co-chaperone family [6]. In the human genome, 49 members of this family with molecular weights ranging from 16–254 kDa have been identified, and all containing a highly conserved J-domain of 69 amino acids located at the N-terminal region which is critical for directing and stimulating the ATPase activity of HSP70 chaperone [6, 53, 54]. The interplay between HSP70s and J proteins provides a sophisticated chaperone machinery that assist in maintaining normal protein homeostasis both under normal and noxious conditions [55]. DNAJ are classified as DNAJA, DNAJB and DNAJC based on their organization, substrate specificity, tissue distribution and subcellular localization [6, 56, 57]. DNAJB3, previously known as MSJ-1 was initially identified in mice as a gene involved in spermatogenesis [58] and while its co-chaperone activity is well established [59], its role in metabolic diseases is emerging. Recent findings from our group and others suggested a possible role of DNAJB3 in the pathogenesis of metabolic diseases associated with IR [7–10, 60]. Our interest to elucidate the pathophysiological role of DNAJB3 in glucose metabolism came from our initial observations showing impaired expression of DNAJB3 mRNA and protein in the adipose tissue of obese and diabetic subjects that correlated with increased P-JNK1, enhanced inflammation and persistent ER stress [8, 9, 61, 62]. The decrease in the expression of DNAJB3 in obese and T2D subjects and the restoration of its normal expression by physical exercise are suggestive of a protective role of DNAJB3 against obesity associated metabolic stress. A similar pattern has been reported previously for two other members of the HSR; namely HSP25/27 and HSP72, both of them are significantly reduced in diabetic conditions and their expression was restored by heat treatment and pharmacologically [44, 63–68].

Using 3T3-l adipocytes and C2C12 skeletal muscle cells, we previously demonstrated an *in vitro* role of DNAJB3 overexpression in improving insulin sensitivity and improving glucose uptake by promoting GLUT4 translocation to the plasma membrane [9, 10]. In 3T3-L1 cells, the overexpression of DNAJB3 elicited the PI3K/AKT pathway as assessed by increased phosphorylation of AKT1 and its substrate AS160 at Ser473 and Thr642 residues, respectively [9]. The mechanism by which DNAJB3 enhanced the AKT pathway remains to be elucidated, but a possible protein-protein interaction between DNAJB3 and AKT is not excluded such as the case of HSP27 [69, 70] and HSP90 [71, 72].

Our work making use of biochemical, structural and cellular biology has now shown that there is a direct connection between these ER stress modulators. Our findings demonstrate a direct binding between DNAJB3 and AKT1 as revealed by GST-pull down experiments using whole C2C12 cell lysate that was confirmed by ITC method using purified DNAJB3 and AKT1 protein. The protein interaction data from ITC showed that there is moderate binding (likely transient) between DNAJB3 and AKT1. The docking results then showed that the DNAJB3 is binding between the protein kinase and PKC domains of AKT1. In cell based-assays, the ability of DNAJB3 to reduce tunicamycin-induced ER stress and to stimulate GLUT4 translocation were significantly impaired upon pharmacological inhibition of AKT1 pathway with Wortmannin and Ly294002 as well as in knock down experiments using si-RNA against AKT1. Thus this cross-disciplinary study reveals the potential mechanism linking these 2 players in ER stress response.

## Supporting information

S1 Raw images Contains all the supporting information and figures. (PDF)

S1 File (PDF)
