# Supplementary figures and images for: DNAJB3 attenuates ER stress through direct interaction with AKT
Source: PLoS One. 2023 Aug 18;18(8):e0290340. doi: 10.1371/journal.pone.0290340 (PMC10437922; doi:10.1371/journal.pone.0290340)

**Original Gels and Blots:**

**Figure 1.**

**Figure 1 (a) original**

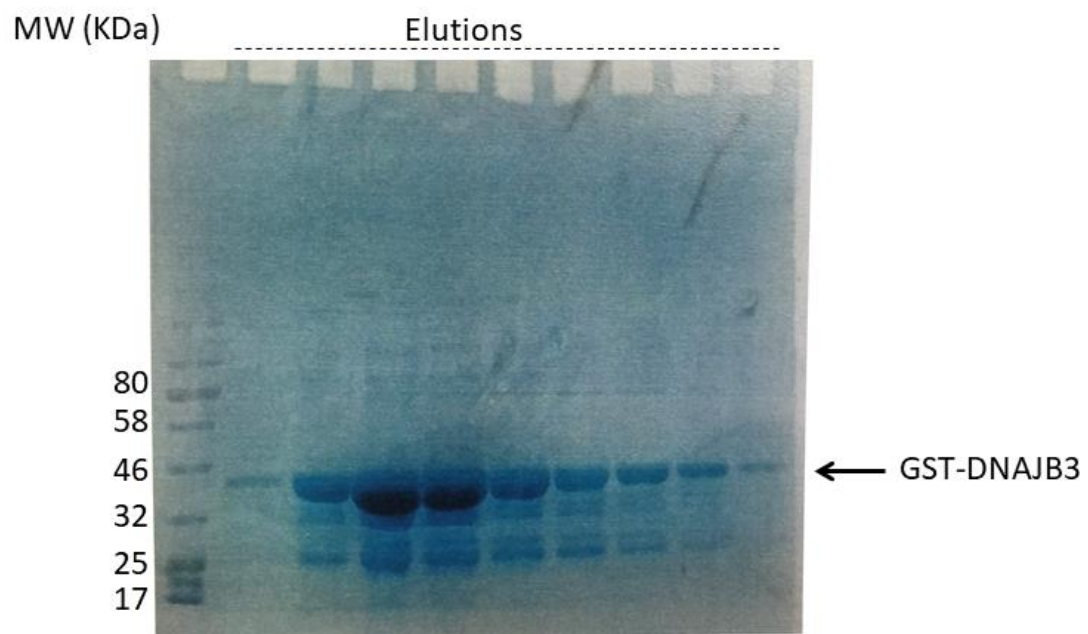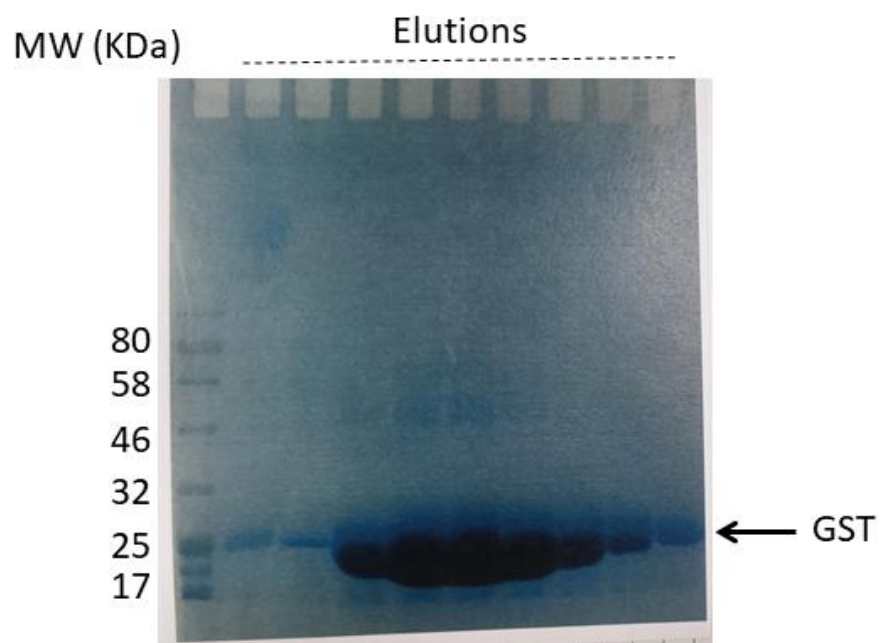

Figure 1 (b) original

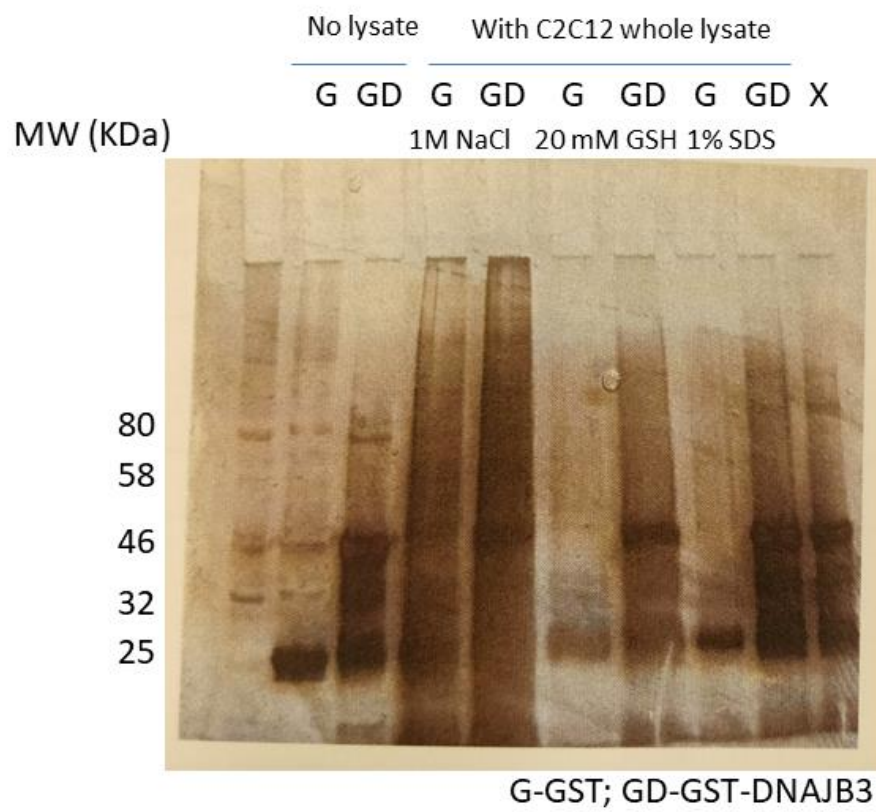

Supplement: S1 Raw images — (PDF) [file pone.0290340.s001.pdf]
