# Supplementary material for: DNAJB3 attenuates ER stress through direct interaction with AKT
Source: PLoS One. 2023 Aug 18;18(8):e0290340. doi: 10.1371/journal.pone.0290340 (PMC10437922; doi:10.1371/journal.pone.0290340)
Supplement: S1 File — (PDF) [file pone.0290340.s002.pdf]

SUPPLEMENTARY INFORMATION

DNAJB3 attenuates ER stress through direct interaction with AKT

Zeyaul Islam[1,#], Abdoulaye Diane[1,#], Mohammed Dehbi[1], Paul Thornalley[1], Prasanna R. Kolatkar[1,*]

[1] Qatar Biomedical Research Institute (QBRI), Hamad Bin Khalifa University (HBKU), Qatar Foundation, Doha, Qatar.

[#] These authors contributed equally to this work

*To whom correspondence should be addressed: Prasanna R. Kolatkar, Qatar Biomedical Research Institute (QBRI), Hamad Bin Khalifa University (HBKU), Qatar Foundation, Doha, Qatar. Tel: +974 445 45889, Fax: +974 445 41770, Email: pkolatkar@hbku.edu.qa

Supplementary Figure 1.

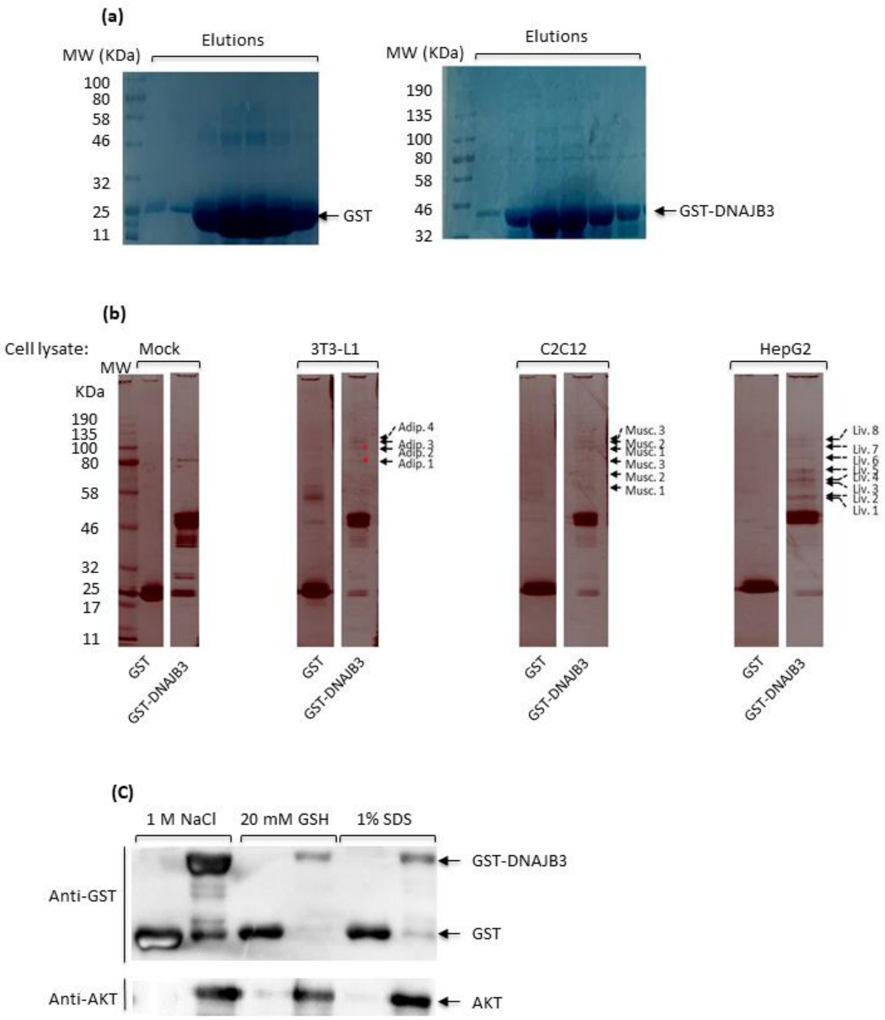

Supp. Figure 1: Full length coommassie staining images of GST and GST-DNAJB3 eluates (a). Representative silver stain images of the specific interacting partners of DNAJB3 after elution with 1% SDS using whole cell lysate from 3T3-L1 adipocytes, C2C12 skeletal muscle and HepG2 liver cells (b). (c) Detection of AKT in 1 M NaCl wash and in elutes as a specific interacting partner of DNAJB3 as revealed by Western blot using ant-AKT antibody. Anti-GST was used as control.

Supplementary Figure 2.

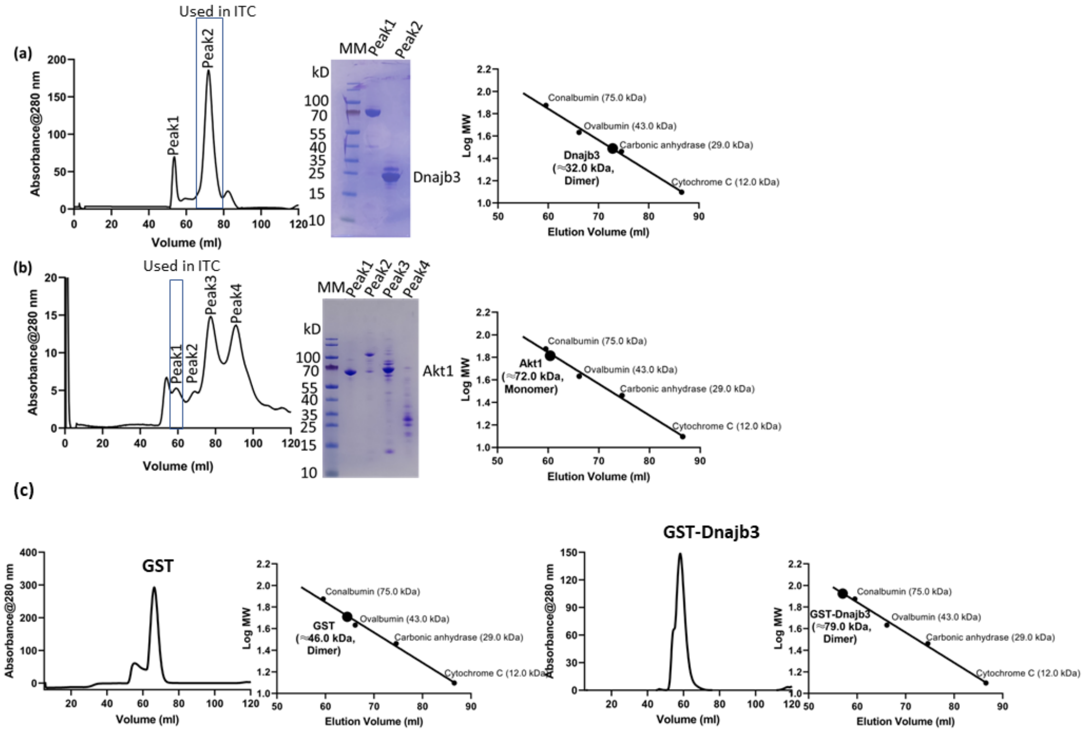

Supp. Figure 2: Purification of DNAJB3 and AKT1 protein. (a) Gel filtration chromatogram of DNAJB3. Affinity purified protein was concentrated and injected into gel filtration column. Protein peaks were run on SDS-PAGE. Peak2 fractions contain all the dimeric DNAJB3 protein. (b) Gel filtration chromatogram of AKT1. Affinity purified protein was concentrated and injected into gel filtration column. Protein peaks were run on SDS-PAGE. Peak1 fractions contain all the monomeric AKT1. (c) Gel filtration chromatography elution profile of GST and GST-Dnajb3.The elution profile of standard molecular masses were plotted along with our proteins to establish the oligomeric status of the proteins.

Supplementary Figure 3.

```
hDnajb3  MVDYYEVLDVPRQASSEAIKKAYRKLALKWHPDKNPENKEEAERRFKQVAEAYEVLSDAKKRDIYDRYGE  70
mDnajb3  MVDYYEVLGVPRQASAEAIRKAYRKLALKWHPDKNPEHKEEAERRFKQVAQAYEVLSDVRKREVYDRCGE  70

hDnajb3  AG-AEGGCTGGRPFEDPFEYVFSFRDPADVFREFFGGQDPFSFDLL-GNPLENILGGSEELLGKQKQSVC  138
mDnajb3  VGEVGGGGAAGSPFHDAFQYVFSFRDPAEVFREFFGGHDPFSFDFFGGDPLENFFGDRRSTRGSRSRG-A  139

hDnajb3  TPFLCLQ---------------------------------------------------------------  145
mDnajb3  VPFSTSFTEFPGFGGGFASLDTGFTSFGSPGNSGLSSFSMSCGGGAAGNYKSVSTSTEIINGKKITTKRI  209

hDnajb3  ---------------------------------                                      145
mDnajb3  VENGQERVEVEEDGELKSLIINGREQLLRINTQ                                      242
```

Supp. Figure 3: Protein sequence alignment of human DNAJB3 (hDNAJB3) and mouse DNAJB3 (mDNAJB3). The identical residues were shown as red, while similar residues as blue. Alignment highlights an extension of approx. 100 amino acids at C-terminal of mDNAJB3.
